# 

**Published:** 1877-01

**Authors:** 


### CONTENTS OF VOL. XXXI.

Address....................494, 9
A Strange Case. Is there a parallel?.................................. 73
Address.................................. 140
A Singular Case in Practice..... 171
Address...........................185, 190
A New Head Rest................ 221
A Case of Salivary Calculus...... 309
A Peculiar Tooth.................... 311
An Obscure Case.................... 312
American Dental Association... 313
American Dental Convention... 314
American Dental Association... 316
Alveolar Abscess.................... 326
Amalgam.............................. 358
A Remarkable Case................ 374
Anæsthetics.......................... 386
A New Microscope................ 400
American Dental Association.... 403
A Case of Suppression of Salivary Secretion......................... 441
A Good Cheap Paste.............. 443
Annual Meeting, Connecticut Valley Dental Association.... 445
Attention............................ 401
Air Chambers........................ 468
A Case of Repeated Excision of the Inferior Dental and Gustatory Nerves for the Cure of Dental Neuralgia................. 516
Biographical.......................... 266
Bibliographical...................... 140
Boston Journal of Chemistry.... 525
Carbolic Acid........................ 26
Camphor and ill-conditioned Wounds............................... 30
Continuous Gum Dentures...... 51
Concerning the Causes of Failures in the use of Cohesive Foil, the Mallet, etc............. 53
Celluloid. What is it?........... 92
Correspondence..................... 131
Commencement.................138, 183
Canada Journal of Dental Science.................................... 226
Connecticut Valley Dental Society.................................... 272
Connection Between Excessive Brain Worry and Bodily Disease.................................. 454
Correction............................ 489
Close of Volume.................... 531
Dental Convention.................. 402
Dental Societies..................... 242
Dental Education.................. 174
Dental Societies..................... 181
Dental Office and Laboratory... 182
Dental Materia Medica and Therapeutics......................... 134
Diameter of the Red Blood-Corpuscles............................... 484
Dental College Education..174, 21
Diseased Gums...................... 229
Disintegration, Arrestation and Preventation of a Recurrence of Caries upon the Proximal Surfaces of the Teeth........... 351
Disinfection by Heat.............. 487
Educational.......................... 446
Eruption of two Inferior Central Incisor Teeth, Second Day After Birth,—Their Extraction Followed by Hemorrhage and Death............................ 378

CONTENTS OF VOL. XXXI.

Erratum... ............................ 271
Eastern Indiana Dental Association.................................. 180
Exposed Pulps, and how Nature Cures Them ................ 69
Experiments with the Turkish Bath............................. . 482
Examiners' Meeting ............. 489
Extracts from an Article on Popular Dentistry......... .... 512
Finishing Fillings..................., 40
Fiat Justitia Again................ 90
Force and its Transmutations... 477
Gingivitcs in Pregnancy...........258
Genuine "Old Fashioned Foil" 139
Good Bye—The Editor Retires. 62
Gold Foil........ ..................... 491
Gold and Temporary Stopping. 346
Grafts of the Dental Follicles.... 473
Hypodermic Injection of Ammonia for Opium Poisoning... 478
Hygrospic Paper ................. 48
How to deprive Iodine of its stain.................................. 485
Hypodermic Injections of Carbolic Acid in Neuralgia........ 27
How to tie a Surgeon's knot.. . 131
Homology of the Dental Tissues, etc .......................249, 338, 358
Hints for Working Celluloid.... 471
Indiana State Dental Association 269
I rise to explain..................... 168
Illinois State Dental Society..... 178
Iowa State Dental Society....... 140
Introductory Lecture, Ohio Dental College.................. 462
Injurious Effects of Vulcanized
Independent Journalism.......... 528
Index of Diseases and their Treatment....................... 530
Rubber................................. 470
Local Anæsthesia.................. 265
Limits of the Surgeon's Responsibility.............................. 125
Minute Subdivision of Coloring Matter....... ..................... 48
Mississippi Valley Dental Association .............. ............ 91
Microscopic ........................... 311
Meeting................................ 139
Merrimack Valley Dental Society.................................... 179
Michigan Dental Society......... 227
Mechanical Dentistry at the Meeting of the American Association.......: ............. 376
Mechanical Dentistry............. 284
Nicotinism......... .................. 444
New Dental Chair.................. 402
Nervousness and Nervines....... 218
Neuralgia..... ......................... 150
Nutrition.................................105
Nerve Stretching in Neuralgia.. 483
Nomenclature......... ............. 489
Osteo Sarcoma........ ........287,
Ohio State Dental Association... 50
Ohio Dental Society............... 490
On Dentine ..................... .... 97
On the Formation of Dentine... 449
Ohio Dental College................ 177
On the Structure and growth of Dentine............................. 361
On the Structure and growth of Dentine ......................... 317
Pulpless Teeth and Alveolar Abscess ................................ 329
Paris Green Poisoning Case.... . 486
Plastic Dentistry.................... 294
Properties of the human Gastric Juice.............................. 481
Proceedings of the Mississippi State Dental Association..... 437
Proceedings of the Northern Ohio Dental Association....... 296
Principles involved in Filling Teeth wtth Gold .............. 239
Pennsylvania State Dental Society........................... ...... 268
Physical Benefit of Sunday... ... 217
Paralysis Treated by Nerve Stretching.................... ...... 136
Principles Involved in the Construction of Artificial Teeth.. 83
Proceedings of the Indiana State Dental Society............... 334
Professional Status and Education.................................. 459
Physiology and Treatment of Dental Pulp. ....................... 507
Quackery............................ 215
Removal ............................... 402
Removing Foreign Bodies from the Æsophagus.................. 484
Rest......................................... 520
Salt for the Throat................ 443
Societies ....................... ...... 265
Southern Dental Association... 271
Statement of a Case................ 223
Saliva Extractor...................... 182
Supreme Court of the United States.................................. 111
Salicylic Acid Mixtures........... 88

Sewer Gases........................... 486
Thirty-first Volume. .............. 49
Treatment of a Suppurating Tooth Socket........................ 45
Treatment of Exposed pulps..... 291
The Principles of Antiseptic Surgery ........................ .... 31
Trans. and Replantation of Teeth 29
Treatment of Teeth during Gestation and Lactation............ 9
The American Leech Trade..... 87
Tooth Ache Remedy............. 88
To Make Leeches Bite............ 89
To the Dental Profession......... 92
The Results of Nerve Injury.... 130
The Anæsthetic Effects of Cold Water...... .................. ..... 137
The Pennsylvania Journal of Dental Science......... ......... 138
The Diamond Disks.........182, 265
Transactions Ohio State Dental Society.............................. 226
Transplantation........................ 214
Transactions Mississippi Valley Dental Association........ 154 200
Tennessee Dental Society........ 270
The Diamond Disk................. 265
Tee Food of the Ancients and of Modern Infants.................. 394
The Principles Involved in the Treatment of Diseased Conditions.............. .................. 273
The Malarial Taint................ 350
Temporary or Plastic Fillings... 391
The Future of Dentistry.......... 384
The Involuntary action of the Nervous System.................. 365
The Unity of Force. Electricity its Superlative Velocity..... 405
Tennessee Dental Association... 414
The Development of Dentine... 423
Tincture Gelseminum.............. 444
Text Books.......... ............... 445
The Physicians' Visiting list.... 445
The Quantity of Drugs used..... 310
The Use of Glycerine.............. 307
Theory of the Action of Iodide of Potassium.......................... 308
The Germ Doctrine and Septicæmia ......... ........................ 480
Tooth Bearing Tumors of the Jaw.................................. 502
The Dangers of Ether............. 524
Use of Tin Foil by Junior Practitioners................................ 447
Vick's Flower and Vegetable Garden......... ..................... 184
Vitalism and Vital Medication.. 259
Vinegar in Opium Poisoning.... 487
Virginia State Dental Association................................. 525
What is Dentistry—the Dignity of the Profession—the Standard to which it should be raised ................................ 410
Wood Rendered Incombustible 219
Who shall Enforce the Dental Law.................. ............. 220
Women in Dentistry...... ........ 100
What's the Matter with Riggs? 526

### Editorial.

### THIRTY-FIRST VOLUME.

This issue of the DENTAL REGISTER is the beginning of the thirty-first volume, and it now enters upon its thirty-first year. In dentistry, as in almost everything else during this time, great changes have taken place; many new things have been produced, and many that then existed have been superseded by improvements; modesof practice have been improved, changed or wholly revolutionized. Changes are not always improvements, nor are substitutes always better than the things they displace, but upon the whole a great advancement has been made in the thirty years just closed. Only those who were familiar with the state of the profession then and have watched its progress during this time up to the present, can fully appreciate what has been accomplished. Let such an one imagine himself placed back thirty years, and call to mind the appliances, instruments, modes of practice and knowledge of that day, and compare with what now exists in these respects then he will be able to appreciate to some extent at least the work that has been accomplished, the progress that has been made.

During this time about seventy dental societies have been organized, and effected an incalculable amount of good. Nine dental colleges have been established. All of which are in active operation, and doing much for the elevation of the profession. During this time six or eight dental periodicals have been established. Some of these are not now published, but they have all, to a greater or less extent, been productive of good.

Now notwithstanding all this, what shall be the future? Is there not much yet to be done?

The successive steps that will be taken in the future of course none can tell; but that advance equal too, if not far greater than hitherto, will be made in the near future, we think no close observer will venture to deny.

To this end the REGISTER, as in the past, will in the future work.

---

### OHIO STATE DENTAL SOCIETY.

### CONTENTS.

COMMUNICATIONS

Treatment of Teeth During Gestation and Lactation . . . . . . 9

Address . . . . . . . . 15

Dental College Education . . . . 21

Carbolic Acid . . . . . . 26

Hypodermic Injections of Carbolic Acid in Neuralgia . . . . . 27

Trans- and Replantation of Teeth . . 29

Camphor in Ill-Conditioned Wounds . 30

The Principles of Antiseptic Surgery . 31

Finishing Fillings . . . . . 40

Treatment of a Suppurating Tooth Socket . 45

Minute Subdivision of Coloring Matter . 48

Hygroscopic Paper . . . . . . 48

EDITORIAL.

Thirty-First Volume . . . . . 49

Ohio State Dental Society . . . 50

Continuous Gum Dentures . . . 51

### THE DENTAL REGISTER,

A MONTHLY MAGAZINE DEVOTED TO THE INTERESTS OF THE DENTAL PROFESSION.

*TERMS REDUCED TO $2.50 PER YEAR. SINGLE COPIES 25C.*

EDITED BY PROF. J. TAFT, D.D.S.

AND

*Published by SPENCER, CROCKER & CO., Ohio Dental Depot.*

The volume commences in January and closes in December.

The REGISTER being issued monthly is always fresh, therefore Indispensable to the practitioner who desires to keep posted up with the new inventions and improvements which are daily developed. Neither expense nor effort will be spared to give value and varie y to its contents. Articles will be illustrated with well executed engravings whenever they will add to the interest or assist to explanation.

Its extensive circulation and frequency of issue make it one of the BEST DENTAL ADVERTISING MEDIUMS in the United States.

All subscriptions must begin with January or July.

Local or special notices, five lines or less, will be inserted for $3 each insertion; over five lines, 50 cts. per line.

All advertising bills payable in advance, or at furthest, after the issue of the first number containing the advertisement. All transient advertisements to be paid for in advance.

☞CONTRIBUTIONS SOLICITED.

Exclusively Editorial Communications should be addressed to Editor.

The latest number of the REGISTER may be ordered with or without other goods at any time, and all letters should be addressed to

*SPENCER, CROCKER & CO., Ohio Dental Depot, Cincinnati, O.*

